# Impact of Proximity and Accessibility of Urgent Care Centers on Emergency Department Utilization for Non-emergent Visits

**DOI:** 10.7759/cureus.82257

**Published:** 2025-04-14

**Authors:** Parnika Telagi, Kevin McGurk

**Affiliations:** 1 Department of Emergency Medicine, Medical College of Wisconsin, Milwaukee, USA

**Keywords:** emergency department utilization, public transportation, social determinants of health (sdoh), transit, urgent care clinic

## Abstract

Introduction

Social determinants of health, including transportation, impact access to healthcare and health outcomes. Urgent Cares (UCs) play an important role in caring for minor injuries and ailments but are asymmetrically distributed and often located in or near more affluent communities. Accessibility of UCs by public and private transit varies widely. While UCs can address acute healthcare needs that do not require emergent treatment, location and transit times may influence their utility. This study aims to characterize the relationship between transit times to different healthcare facilities and ED utilization for non-emergent visits.

Methods

Publicly available ED utilization data was obtained for Milwaukee County from 2019-2020. ED visits by ZIP code were categorized as either emergent or non-emergent as defined by the validated New York University - Emergency Department Visit Algorithm (NYU-EDA) classification system. A Google Maps application programming interface was used to compute the public and private transit times between the population center of each census tract and its nearest UC and ED. Associations between transit times and the percent of non-emergent ED utilization were measured.

Results

Median transit times were shorter to EDs than UCs across the county. Decreased transit times to EDs were associated with increased ED utilization for non-emergent complaints across both private and public transportation methods (p=0.23 and p=0.02, respectively). Increased transit time to UCs was associated with increased ED utilization for non-emergent complaints across private and public transportation (p=0.07 and p=0.34).

Conclusions

Decreased transit times to EDs and increased transit times to UCs are associated with increased ED utilization for non-emergent complaints. This suggests accessibility and geographic proximity may play a role in patient choices regarding where to seek care and carries public health and healthcare policy implications.

## Introduction

Socioeconomic status (SES) is an essential social determinant of health and individuals with lower SES encounter more barriers to healthcare access than those with a higher SES [1]. The availability of reliable transportation is closely correlated with SES and represents another important social determinant of health [2]. Limited access to transportation can lead to worse clinical outcomes and increased emergency department (ED) utilization [3]. In the absence of reliable or timely public and private transit options, patients may even require emergency medical services for non-emergent transportation. 

Urgent care (UC) centers are a commonly used alternative to EDs for patients with minor injuries and low severity ailments. The number of UCs has grown rapidly in recent years and they are often preferred by patients for cost or convenience and may help mitigate against ED crowding when appropriately utilized [4-7]. Some evidence suggests UC use may prevent subsequent unnecessary ED utilization [8]. The utility of UCs though is incumbent upon these centers being accessible to patients seeking care.

Prior studies have demonstrated an association between UC locations and more affluent communities and higher rates of private insurance [9]. Research has also examined the effects of hospital location and primary care utilization with ED visits for non-emergent healthcare needs [10]. However, the role of geographic proximity and accessible transportation to UCs and its impact on ED use is largely unknown. This study seeks to characterize the relationship between proximity to and accessibility of UCs and emergency department utilization for non-emergent complaints.

## Materials and methods

The study was conducted in Milwaukee County, the most populous county in Wisconsin and home to more than 900,000 residents in geographically and socioeconomically diverse communities. Publicly available data from the Milwaukee Health Care Partnership was used to obtain the number of non-emergent and emergent ED visits as defined by the validated New York University - Emergency Department Visit Algorithm (NYU-EDA) classification system [11-13]. The NYU-EDA is a model that uses discharge diagnoses to classify ED visits by broad categories including non-emergent, emergent but preventable if appropriate ambulatory care had been received, and emergent and not avoidable. Visits were categorized by the ZIP code of patient addresses within Milwaukee County for each quarter from January 1, 2019 through December 31, 2020, the last year with available data. Non-emergent ED visits were divided by the total number of ED visits per ZIP code to calculate the percentage of non-emergent care.

Urgent care centers were included when meeting criteria as defined by the Urgent Care Association of America [14,15].

For a more granular assessment of transit times to relevant healthcare facilities across the county, census tracts were used. Census tracts are smaller subdivisions of a county with a typical population size of around 4,000. The center of population for each census tract as defined by the U.S. Census Bureau was recorded as a set of coordinates [16]. Google Maps was then used to compute the transit time via public and private transit from these coordinates to nearby health centers. Public transportation options within the community include bus lines and limited streetcar routes. An application programming interface (API) was coded to capture the shortest commute from each census tract to its nearest UC and ED. As there are several census tracts within a ZIP code, the median transit time between the census tracts of each given ZIP code was used. To ensure representative travel times for real commuting conditions, the API was run for several times of day and days of the week.

To characterize any association between these results and the SES of each community, the social vulnerability index (SVI) was used [17]. The SVI is a public health tool quantified by the US Centers for Disease Control and Prevention (CDC) that measures numerous social and economic characteristics at the census tract level. Scores range from 0 to 1, with a lower SVI indicating less vulnerable communities and higher scores for more socioeconomically disadvantaged communities.

In order to assess for suspected changes in ED visit patterns following the onset of the COVID-19 pandemic, a Chi-squared Proportion Test was performed. The relationship between transit times and ED utilization was calculated using the Pearson Correlation Coefficient. Statistical analyses were performed using the R software environment (R 4.2.3; R Foundation for Statistical Computing, Vienna, Austria).

This study was declared exempt by the Medical College of Wisconsin Institutional Review Board.

## Results

Milwaukee County includes 13 EDs and 10 UCs. Locations with an ED had a higher median SVI than those with UCs (0.63 vs 0.49, respectively). The median commute times to EDs were typically shorter than to the closest UCs. Median total commuting time to the nearest UC and ED via public transportation were 31 minutes (IQR=12) and 22 minutes (IQR=13), respectively (*p < *0.001). Commutes were faster with private transit, with median UC and ED commute times of 9 minutes (IQR=5) and 7 minutes (IQR=4), respectively (*p *< 0.001).

**Figure 1:**
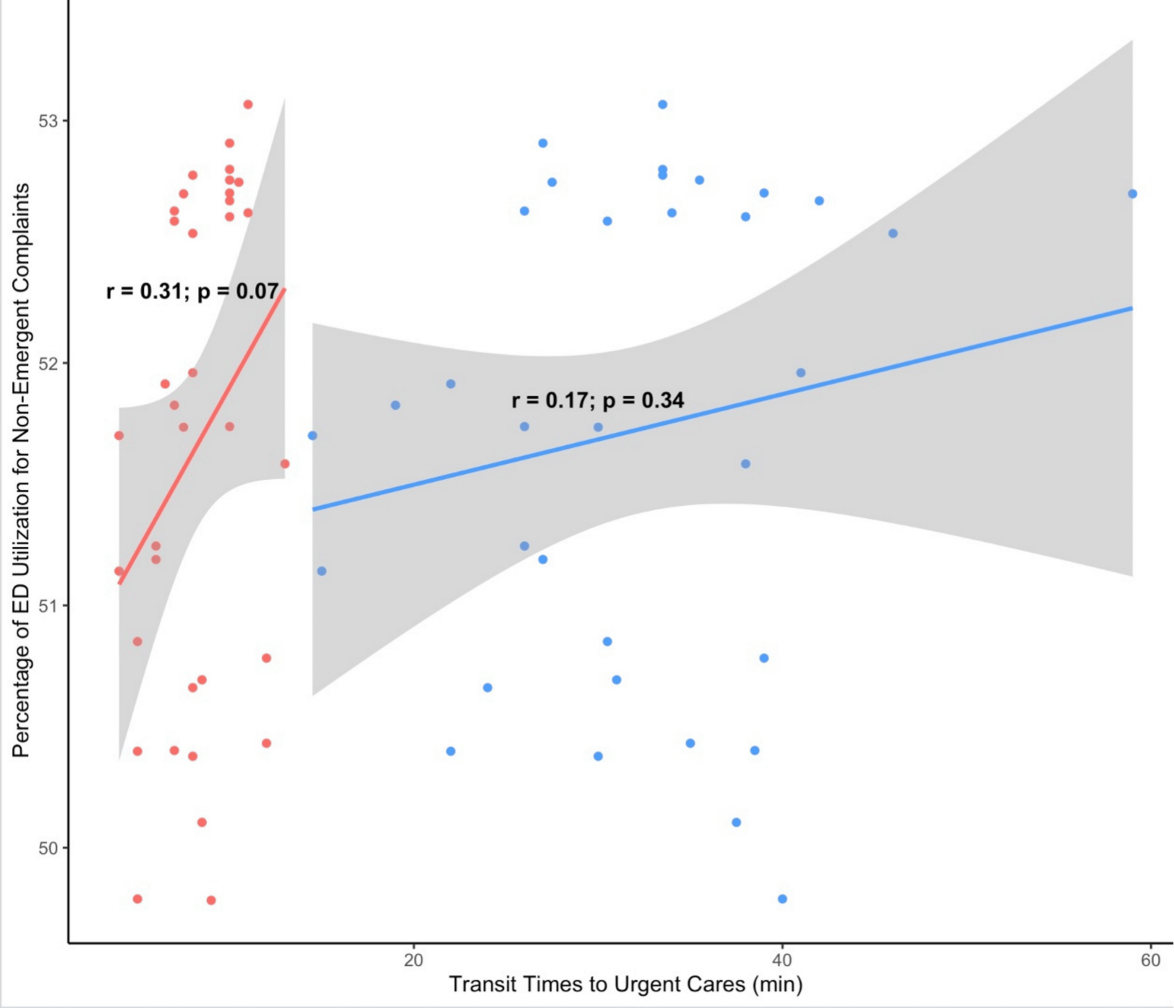
Urgent Care Transit Times and Non-emergent ED Visits Blue: Public Transit Times Red: Private Transit Times

As transit times increased to the nearest UCs, the percentage of ED visits for non-emergent complaints trended upwards as shown in Figure 1.

**Figure 2:**
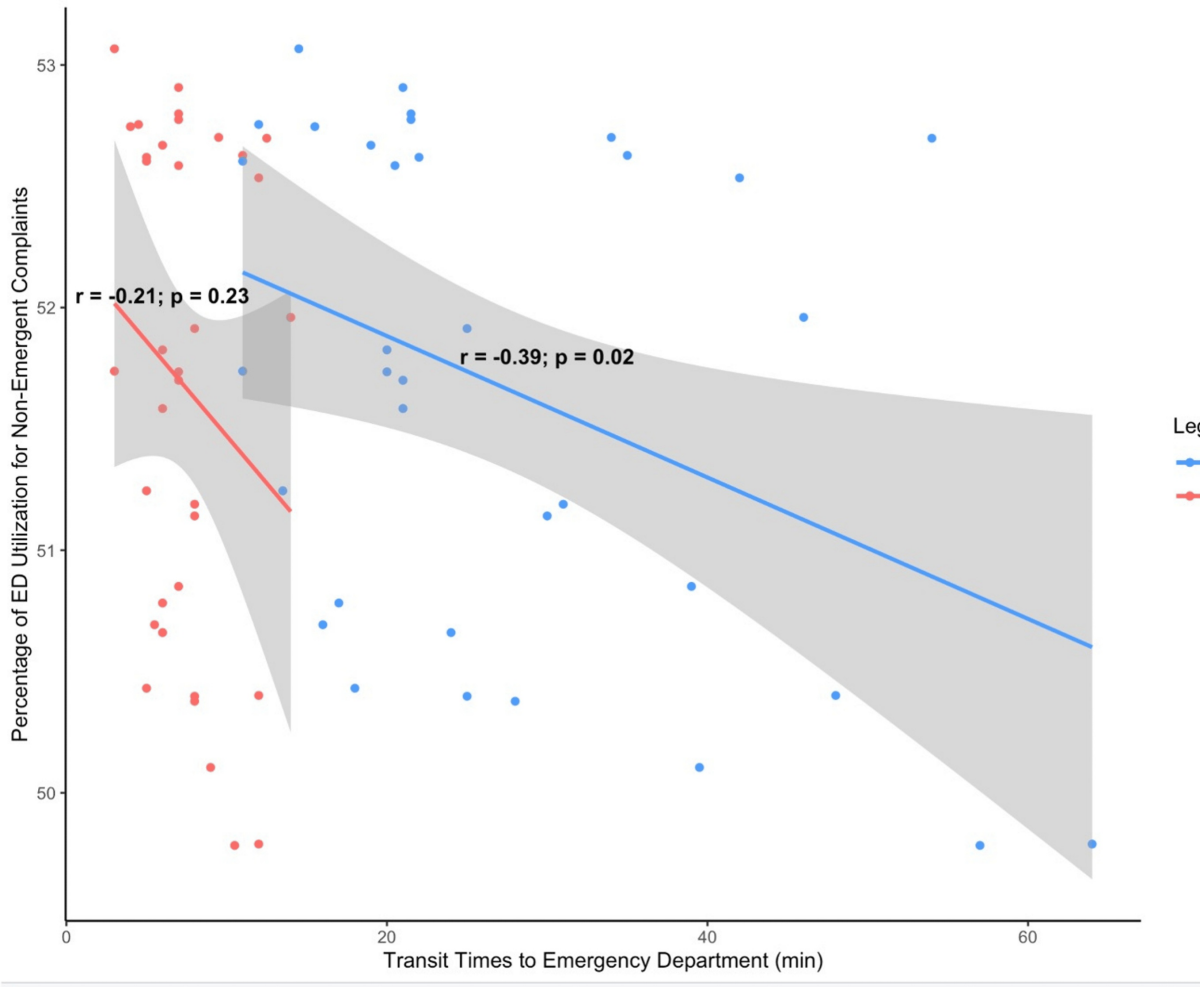
ED Transit Times and Non-emergent ED Visits Blue: Public Transit Times Red: Private Transit Times

This trend was similar for both public and private transportation, though neither reached statistical significance. As commute times to the nearest ED increased though, hospital utilization for non-emergent complaints trended downward as shown in Figure 2. This was true for both public and private transit times though the relationship was only statistically significant for public transit times.

**Figure 3:**
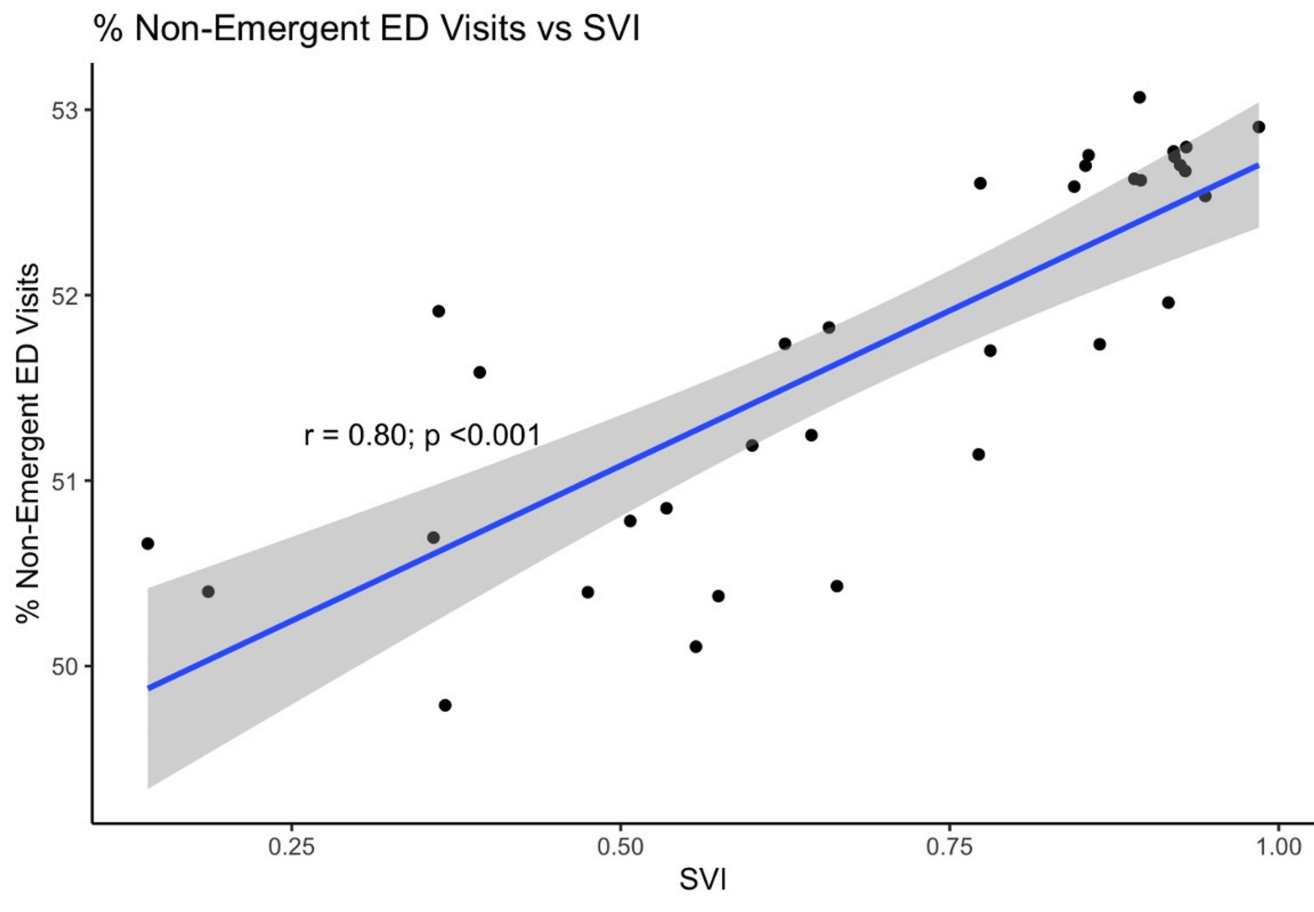
Social Vulnerability Index (SVI) and Non-emergent ED Visits

The median SVI for each zip code was strongly correlated with ED utilization patterns as shown in Figure 3. As the social vulnerability of a community increased, the percentage of non-emergent ED visits for individuals in that community also increased. Conversely, more affluent zip codes had proportionately fewer non-emergent hospital visits. 

When evaluating for an impact on utilization trends related to the COVID-19 pandemic, there was a small but statistically significant decrease (52.36% vs 52.07%, p= 0.02) in non-emergent ED visits after the onset of the pandemic during the last three quarters of 2020 as seen in Table 1. 

**Table 1:** Percentage of Non-emergent ED visits Pre- and Post-COVID-19 Pandemic

	Pre-COVID-19 Period	Post-COVID-19 Period
Non-emergent Visits	298908	109179
Emergent Visits	271956	100512
Non-emergent ED Visits	52.36%	52.07%

## Discussion

Though "accessibility" could entail a number of elements, including patient- or facility-specific factors such as visit costs and insurance copays, this study examines access to acute care health facilities through the narrower lens of transit and geographic proximity. While not all statistically significant, there are general trends among our results demonstrating that increased public and private transit times to EDs and decreased transit times to UCs are both associated with less ED utilization for non-emergent complaints. In particular, increased public transit commute times to the nearest hospital had the strongest negative relationship with non-emergent ED utilization. As even our statistically significant results reflected weak associations, findings must be interpreted with caution. However, the pattern supports what many might have presumed: when it is easy to get to an urgent care, patients are less likely to go to the hospital for needs that could otherwise have been addressed at UCs. Similarly, communities with less expedient access to UCs are more likely to turn to the ED for non-emergent complaints. These findings have implications for how health systems target ED overcrowding and reinforce the importance for those involved in healthcare administration and public policy to consider the vital role transportation plays in dictating how and where patients seek care [18-20].

Perhaps unsurprisingly, SVI was also closely correlated with non-emergent ED utilization. More socioeconomically disadvantaged communities used the hospital for health concerns that potentially could have been addressed in alternative settings, including primary care clinics and UCs. These results reaffirm prior studies addressing how disadvantaged populations use the ED [21,22]. Notably, though, our study also demonstrates that UCs are generally located in more affluent communities as compared to EDs. The disparate socioeconomic characteristics of communities with UCs vs EDs represents a structural factor that likely contributes to variations in non-emergent healthcare utilization practices.

Importantly, the study period in question also represents a highly atypical time in medicine. The early stages of the COVID-19 pandemic dramatically altered the healthcare landscape. Comparisons made across each quarter included (eight total) found no demonstrable differences in trends with respect to geography or transit times. The cumulative post-COVID-19 period, defined for this study as the last three quarters of 2020, had substantially fewer ED visits as compared to the pre-COVID-19 period. However, non-emergent ED utilization as a percentage of all ED visits was minimally impacted. The relatively unchanged proportion of non-emergent visits despite a sizable drop in total ED visits may reflect a commensurate decrease in UC hours or primary care availability during the pandemic but merits further study.

Limitations

This study is not without limitations. It is a retrospective analysis and conducted in one primarily urban and suburban county. In areas with different demographic compositions or varying public transit options and accessibility, the results may not be uniformly generalizable and replication in different locales is warranted. Additionally, ED use patterns were measured at the ZIP code level. While our study took steps to better reflect more localized variation by incorporating census tracts, this may not adequately capture the significant variation in transit times that can occur over small areas. Furthermore, this study relied upon aggregate hospital utilization data that could not be stratified by demographics such as age and gender. Unmeasured variation in community composition could also contribute to differences in healthcare utilization. Lastly, this study includes time periods prior to and then following the onset of the COVID-19 pandemic. While pre- and post-pandemic comparisons were made, the unique impact of that pandemic may limit the generalizability of our results. 

## Conclusions

Socioeconomic vulnerability is strongly correlated with ED utilization for non-emergent healthcare needs. Public and private transit accessibility to the nearest health centers also impacts ED utilization patterns. Longer public transit commutes to the nearest emergency department are associated with fewer hospital visits for non-emergent complaints. These findings reinforce the importance of transportation as a social determinant of health and the role of geographic proximity in healthcare utilization.
